# Results of the Italian cross-sectional web-based survey “Nutrition and breast cancer, what would you like to know?” An attempt to collect and respond to patients’ information needs, through social media

**DOI:** 10.3389/fonc.2024.1436610

**Published:** 2024-09-25

**Authors:** Greta Caprara, Eleonora Pagan, Lucilla Titta, Maria Tieri, Giada Magionesi, Silvia Gallosti, Vincenzo Bagnardi, Ketti Mazzocco, Manuelita Mazza

**Affiliations:** ^1^ Department of Experimental Oncology, IEO, European Institute of Oncology IRCCS, Milan, Italy; ^2^ Department of Statistics and Quantitative Methods, University of Milano-Bicocca, Milan, Italy; ^3^ Applied Research Division for Cognitive and Psychological Science, IEO, European Institute of Oncology IRCCS, Milan, Italy; ^4^ Department of Oncology and Hemato-Oncology, University of Milan, Milan, Italy; ^5^ Division of Medical Senology, IEO, European Institute of Oncology IRCCS, Milan, Italy

**Keywords:** breast cancer, the internet, social media, diet, nutrition, foods, supplements, health care professionals

## Abstract

**Introduction:**

Several studies have demonstrated that, following a breast cancer (BC) diagnosis, patients are eager to obtain information on cancer and nutrition, in order to ameliorate both their quality of life (QoL) and disease outcome. To avoid BC survivors to get wrong information from unreliable sources, healthcare providers need to be aware of patients’ needs, to guide them toward optimal nutrition recommendations, aimed at preventing tumor recurrence and increasing survival rates.

**Material and methods:**

The cross-sectional web-based survey “Nutrition and breast cancer, what would you like to know?” has been conceived and conducted, in Italy, between the 2nd and the 25th of June 2023. The link to the 19-items questionnaire, structured in 6 sections, was distributed *via* social media (Facebook and Instagram), newsletter, institutional websites, and printed flyers. Patients’ responses were collected and analyzed, reporting absolute and relative frequencies.

**Results:**

A total of 1616 participants (98.9% female and 1.1% male), with an average age of 47.5 years, answered the survey. Only subjects who declared having previously received a BC diagnosis (N=1159, 71.7%) were included in the present analysis. Overall, the respondents showed a wide interest in understanding whether nutrition might help to manage therapy side effects, as well as knowing how specific diets, foods, nutrients, and supplements could affect disease onset, progression and prognosis. Importantly, the need to receive evidence-based information from the “referring physician/specialist” and “nutritionist/dietitian” was expressed by 95.8% and 88.8% of them, respectively.

**Discussion:**

In this study, we primarily aimed at intercepting nutrition information needs and sources of an Italian BC survivors’ group. Based on that, we first organized a proactive digital intervention, to respond *via* Instagram live broadcasts to patients’ “cancer and nutrition”-related questions. Secondly, we arranged a healthcare providers dedicated-workshop focused on the latest evidence-based knowledge on nutrition and BC. It is crucial, in fact, that once healthcare professionals capture patients’ information needs, they can respond with appropriate nutritional guidance, counseling and education programs, while counteracting misleading and incorrect messages.

## 1 Introduction

In 2022, the global cancer burden has risen to around 20 million cases worldwide and this number is projected to increase to about 35.3 million in 2050 (1). Breast cancer (BC) is the second most commonly diagnosed tumor, with an incidence and a 5-year prevalence rate of 11.5% and 15.3%, respectively (1). In Italy, instead, BC is the most frequently occurring cancer, accounting for 13.2% of all new identified malignancies and recording the highest incidence among women (28.2%) (1). However, in 2022, the estimated survival rate at 5 and 9 years after diagnosis was 88% and 91%, respectively (2). Nowadays, 834.200 women, corresponding to 1.4% of the total population, are estimated to live in Italy after a BC diagnosis (2).

Therefore, it would seem quite evident that an increasing amount of people is living with this disease even for many years, often facing therapies frequently accompanied by severe side effects (such as weight gain; abdominal and subcutaneous swelling; nausea and vomiting; fatigue; loss of appetite; changes in taste; diarrhea or constipation etc.) (3–6). Accordingly, a growing amount of evidence revealed that, after diagnosis, health, quality of life (QoL) and psychological concerns are extremely common among BC survivors, who often feel their information needs are not fulfilled enough by physicians (7–10). Diet and nutrition tasks are some of the major worries of patients, who might express a strong need of information to ameliorate post‐treatment side effects, their QoL, to meet specific nutritional needs, fight cancer or prevent its recurrence (9, 11–13).

Unlike in the past, when patients relied on their physician as the major source of information and recommendations, nowadays people are more rapidly accessing information through the media and, particularly, the Internet and social networks (14–18). Indeed, this is happening also with cancer patients and especially BC survivors, 10% to 43% of whom use the Internet to search for information (10, 19–23). Given the abundance of misreporting found in the media and online, BC patients are at high risk of misinformation and, as a consequence, they could be persuaded to follow messages, judged trustworthy and accurate, which are, instead, false or misleading (10, 24, 25). Much of that misinformation is related to diets, foods, food components or dietary supplements (26–29), which very often are deemed to be able to cure, cause cancer or possess special health benefits (30, 31).

Nowadays, there is good and reliable evidence that a diet rich in vegetables, fruits, legumes, wholegrains and limited in energy-dense foods, red and processed meat, sugar sweetened drinks and alcohol may reduce tumor risk (32, 33), improve prognosis (34–36), and promote wellness during the cancer continuum (34–36). On the other hand, with the exception of alcohol, whose consumption is strongly associated with an increased risk of breast cancer (33, 37), there are no authoritative evidence-based recommendations to consume or avoid particular foods, nutrients, or food constituents in order to prevent or cure BC (6, 33, 37–45). Therefore, the latest evidence available from the most reliable institutions, such as the World Cancer Research Fund (WCRF/AICR) and the American Society of Clinical Oncology (ASCO), has established that following the advice for general cancer prevention could be appropriate both before and after a BC diagnosis (33, 36–40, 46–48). However, even if not strong enough to make specific recommendations, emerging limited but suggestive evidence links (i) a healthy body weight, (ii) being physically active, (iii) eating foods containing fiber, (iv) eating foods containing soy and (v) a lower intake of total fat (in particular saturated fat), with a better prognosis for BC patients (39, 40).

Although both the Internet and social media could potentially share and spread misinformative or biased nutrition and health content, they also have the potential to provide support and even transmit the right messages to BC patients and their caregivers (49–58).

In this context, we have conceived and conducted the online survey “Nutrition and breast cancer, what would you like to know?”. We first aimed at intercepting Italian BC survivors’ nutrition information needs and current sources of advice, as well as, ascertain where they would rather find reliable information, in the future. According to that, we developed *ad hoc* interventions aimed at giving patients reliable nutrition and lifestyle recommendations, through their preferred communication channels. Specifically: (i) during Breast Cancer Awareness Month, we organized Instagram live broadcasts intended to answer to “cancer and nutrition”-related questions; (ii) we arranged and planned a healthcare providers dedicated-workshop focused on the latest evidence-based knowledge on nutrition and BC. Indeed, it is crucial for healthcare professionals to understand patients’ information needs in order to provide appropriate guidance, counseling, and education programs. This will help to prevent the dissemination of distorted and flawed messages that could hinder adherence to optimal nutrition and lifestyle recommendations aimed at preventing tumor recurrence and increasing survival rates.

## 2 Materials and methods

### 2.1 Study design and respondents

The survey “Nutrition and breast cancer, what would you like to know?” was conceived, designed, reviewed by a group of experts from various disciplines (including oncology, nursing, dietetics, nutrition, science communication, and psychology), and approved by the European Institute of Oncology (IEO), IRCCS, Milano, Italy. Criteria for inclusion comprised: (i) adults ≥18 years of age; (ii) resident in Italy; (iii) subjects’ willingness to participate, after having given consent by mandatory selecting the first question: “*I voluntarily agree to complete the questionnaire, without providing any data allowing my direct identification, and I am aware that the information here provided will be used for scientific dissemination and research purposes only*”. Exclusion criteria were the following: (i) unable and/or unwilling to complete the questionnaire. Participants were provided with all the information in writing and they reserved unconditional or absolute right of withdrawal at any time and without giving any reason. Participants were not offered any compensation or incentives for participation.

Data have been processed under the European Union General Data Protection Regulation (EU 2016/679).

### 2.2 Survey design

The anonymous, voluntary, online, cross-sectional questionnaire “Nutrition and breast cancer, what would you like to know?” was distributed in Italy, from the 2nd to the 25th of June 2023, using Microsoft Forms (Microsoft Corporation, Redmond, WA, USA). Participants were recruited through Smartfood Program (59) and IEO (60) social media (Facebook and Instagram), patients’ social network groups, healthcare professionals’ social media pages, newsletter, institutional websites and printed flyers. In order to achieve the largest possible sample, within the selected timeframe, respondents could also share the survey link with their acquaintances.

This 19-items questionnaire aimed at understanding respondents’ “nutrition and cancer” information needs and current sources of advice, as well as ascertaining where they would rather find reliable information in the future. It consisted of six sections: (i) demographic characteristics and information sources; (ii) management of cancer treatment side effects; (iii) specific diets; (iv) foods and nutrients; (v) dietary supplements and (vi) uncertainty management. The latter is the Italian translation of the short version of the Intolerance of Uncertainty Scale (61).

The full English translated version of the questionnaire is provided in the Supplementary materials as **Supplementary Data Sheet 1**.

### 2.3 Statistical analysis

Categorical variables were represented with absolute and relative frequencies, while continuous variables were reported by mean and standard deviation.

The Pearson’s Chi-square test was used to compare the percentages of responses categorized as “Sometimes/Often/Always” versus “Never/Rarely” across different age classes for the two questions related to patients’ sources of information.

Analyses were performed with SAS software v9.4 (SAS Institute, Cary, North Carolina, USA).

## 3 Results

Overall, 1616 questionnaire answers were collected. In the present analysis we only included the 1159 respondents who declared having previously received a BC diagnosis (71.7% of the total sample). Among the excluded there were: 85 health professionals (5.3%); 166 caregivers, relatives, parents, spouses/partners of people diagnosed with BC (10.3%); 114 friends of a person diagnosed with BC (7.0%); and anyone else who selected the “other” option (5.7%).

Of the 1159 respondents considered, 1021 (88.1%) heard about this questionnaire through the Smartfood Program (59) and/or the IEO (60) communication channels (social networks, newsletters and websites), 6 (0.5%) through flyers on hospital wall, and 132 (11.4%) through word of mouth. At the end of the questionnaire, 34.2% of the 1159 respondents stated they would like to know more about “nutrition and breast cancer”.

Detailed demographic characteristics of the 1159 respondents (99.5% female and 0.5% male), whose mean age was 49.4 years, are shown in **Table 1**.

**Table 1:** Sociodemographic characteristics of survey respondents (N = number of respondents, % = percentage of respondents; total respondents N = 1159).

	N	%
Age class (years)		
<40	161	13.9
40-49	426	36.8
50-59	417	36.0
≥60	155	13.4
Mean (Standard deviation)	49.4 (8.8)	
Sex		
Female	1153	99.5
Male	6	0.5
Home country area		
North	625	53.9
Center	206	17.8
South + Islands	328	28.3
Education		
Junior high school	68	5.9
High school	573	49.4
Master’s degree	393	33.9
Postgraduate degree	125	10.8

### 3.1 Patients’ information needs regarding “quality of life and nutrition in oncology”

As already demonstrated by many studies, BC patients are particularly eager for nutrition information (9, 11–13). In order to avoid them getting wrong advices from unreliable sources, it is crucial that healthcare providers, once aware of patients’ needs, are able to guide them toward optimal nutrition recommendations, aimed at increasing the chance of survival and lead a better QoL.

In order to intercept nutrition information needs and sources, we first asked the respondents where they currently look for information about “quality of life and nutrition in oncology”.

In this section, the categories “Sometimes”, “Often”, and “Always”, as well as “Moderately”, “Quite a bit”, and “Extremely”, have been consolidated to simplify the presentation of results and emphasize the most significant trends in patient attitudes. However, figures and supplementary tables present the data for each level of the variables analyzed.

**Figure 1:**
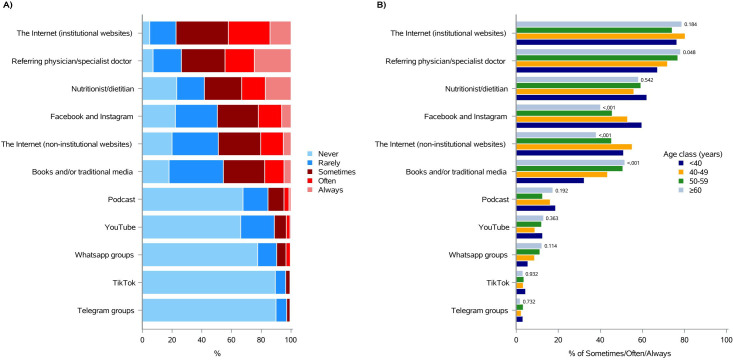
Information sources currently consulted about “quality of life and nutrition in oncology”. **(A)** Total distribution; **(B)** age class distribution (<40, 40-49, 50-59, ≥60).

As illustrated in **Figure 1** and **Supplementary Table 1**, most of them (77.3%) “sometimes, often or always” look for information on the Internet (institutional websites), followed by the referring physician/specialist doctor (73.8%), nutritionist/dietitian (58.3%), Facebook and Instagram (49.4%), the Internet (non-institutional websites) (48.8%), and books and/or traditional media (45.5%). Podcasts, YouTube, Whatsapp groups and TikTok were used by only the 15.3%, 11.0%, 9.7% and 3.5% of the sample, respectively. Telegram groups, instead, were chosen only by the 2.7% of subjects (**Figure 1** and **Supplementary Table 1**).

Overall, it seemed that age did not particularly affect respondents’ source choice, except for Facebook and Instagram, which were used “sometimes, often or always” by 59.6% of patients under 40 years, compared to 40.0% of patients over 60 years (p-value<0.001; **Figure 1B** and **Supplementary Table 1**). In agreement with this, books and/or traditional media were accessed “sometimes, often or always” by the 51.6% of patients aged 60 or more, compared with the 32.3% of those under 40 (p-value <0.001; **Figure 1B** and **Supplementary Table 1**).

Providing correct and effective information it is crucial for delivering high quality patient-centered care (9). We then wanted to find out where respondents would prefer to find reliable information about “quality of life and nutrition in oncology” in the future.

**Figure 2:**
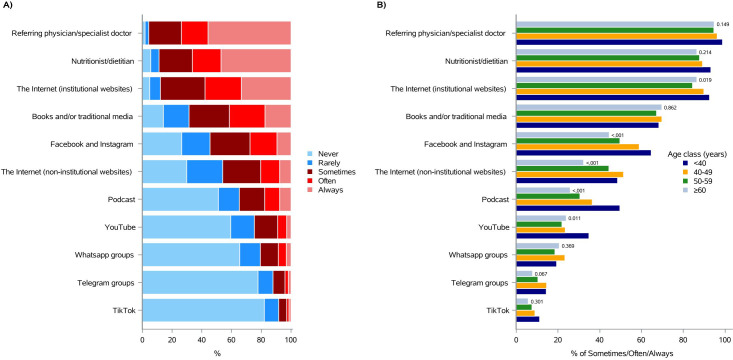
Preferred sources for receiving information, in the future, about “quality of life and nutrition in oncology”. **(A)** Total distribution; **(B)** age class distribution (<40, 40-49, 50-59, ≥60).

As shown in **Figure 2** and **Supplementary Table 2**, the 95.8% of the respondents would like to have information, “sometimes, often or always”, from the referring physician/specialist doctor, followed by the nutritionist/dietitian (88.8%), the Internet (institutional websites) (87.8%), books and/or traditional media (68.6%), Facebook and Instagram (54.4%), and the Internet (non-institutional websites) (46.0%). Podcasts, YouTube, Whatsapp groups, Telegram groups and TikTok were selected by the 34.7%, 24.4%, 20.6%, 12.0%, and 8.3% of patients, respectively (**Figure 2** and **Supplementary Table 2**).

Consistently with the previous question responses, age did not essentially influence patients’ answers; however, the 64.6% of subjects under 40 chose Facebook and Instagram, “sometimes, often or always”, as source of information, while only the 44.6% of those over 60 would use these social networks (p-value <0.001; **Figure 2B** and **Supplementary Table 2**). Interestingly, Podcast, an emerging audio information resource, would be chosen by almost the 50% of respondents under 40, while only the 25.8% of the over 60 would select it (p-value <0.001). Similar differences have been observed with Telegram (14.3% *versus* 7.7%, p-value = 0.067) and TikTok (11.1% *versus* 5.8%, p-value = 0.301) (**Figure 2B** and **Supplementary Table 2**).

### 3.2 Patients’ information needs regarding the effects of specific diets, foods, nutrients and dietary supplements in managing cancer treatment side effects

**Figure 3:**
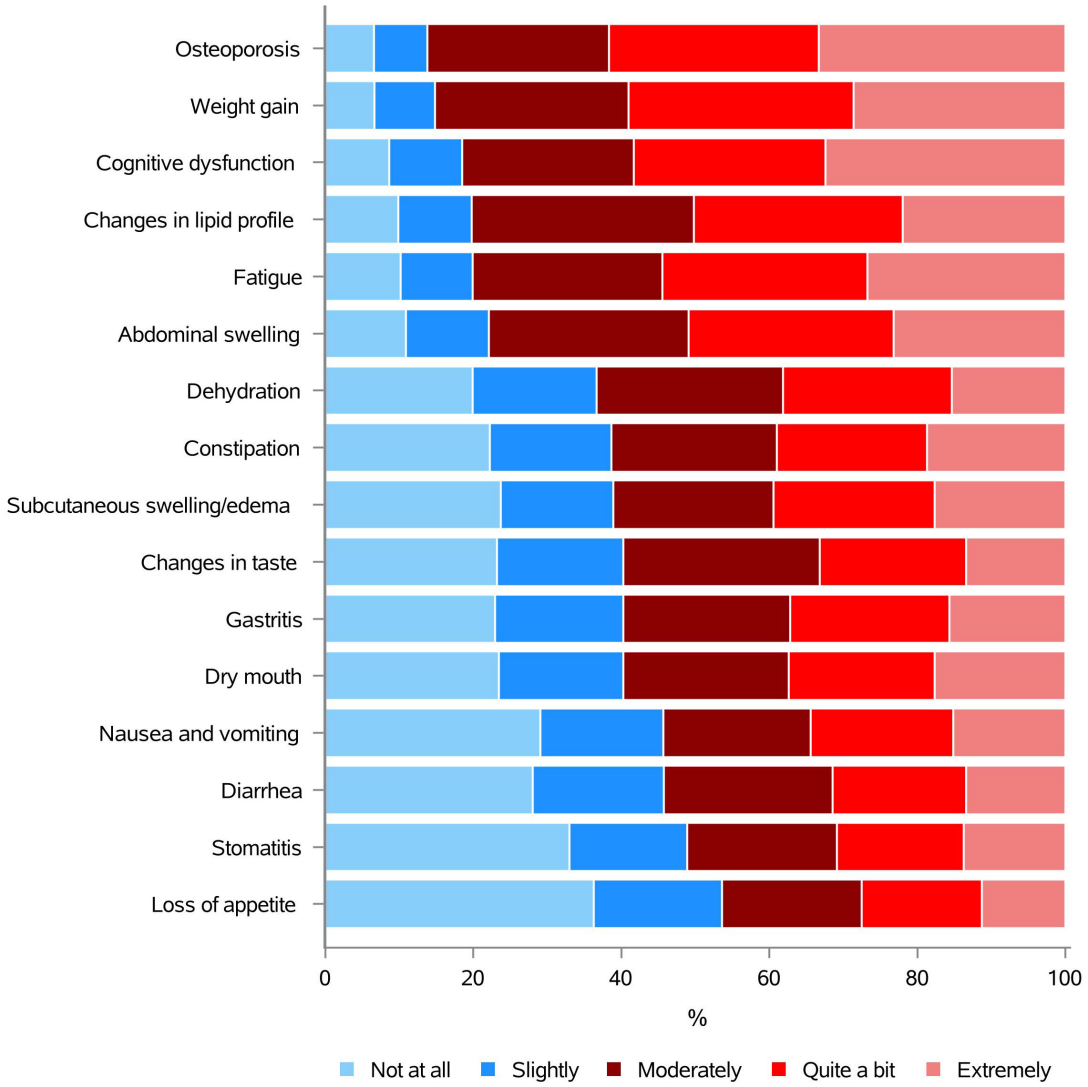
Information needs concerning the effects of specific diets, foods, nutrients and dietary supplements in managing cancer treatment side effects.

Mounting evidence showed that people diagnosed with BC often express the need to obtain nutrition information related to cancer treatments and their side effects (62). Accordingly, most of the respondents (86.2%) were seeking “moderately, quite a bit or extremely” information on osteoporosis, followed by weight gain (85.1%), cognitive dysfunction (81.5%), changes in lipid profile (80.2%), fatigue (80.0%), and abdominal swelling (77.9%). Dehydration (63.4%), constipation (61.3%), subcutaneous swelling/edema (61.1%), taste changes (59.8%), gastritis (59.7%), dry mouth (59.7%), nausea and vomiting (54.3%), diarrhea (54.3%), stomatitis (51.1%), and loss of appetite (46.4%), were also selected by almost the 50-64% of our sample (**Figure 3** and **Supplementary Table 3**).

### 3.3 Patients’ information needs regarding the use of specific diets, in oncology

**Figure 4:**
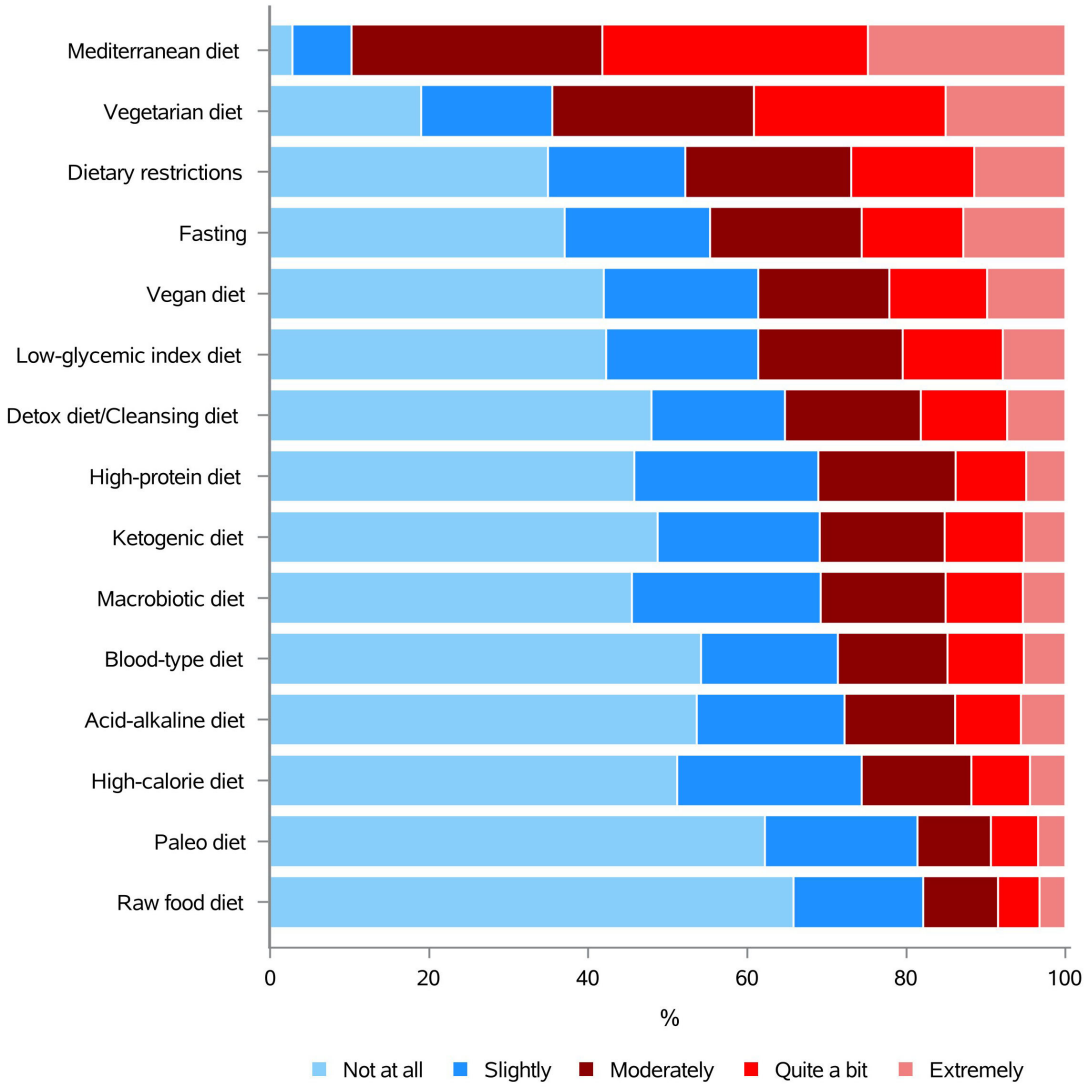
Information needs concerning the use of specific diets, in oncology.

BC patients are very commonly concerned about the best diet to follow after diagnosis and they often express the need for information on this topic, to possibly ameliorate their health (9). The answers to question 14 “How much do you need to know about the use of the following diets in oncology?” showed patients were mostly interested in knowing more about Mediterranean diet (89.7%), followed by vegetarian diet (64.6%), dietary restrictions (47.8%) and fasting (44.8%). Vegan diet (38.6%), low-glycemic index diet (38.7%), detox diet/cleansing diet (35.3%), high-protein diet (31.1%), ketogenic diet (30.9%), macrobiotic diet (30.7%), blood-type diet (28.7%), acid-alkaline diet (27.8%), high-calorie diet (25.6%), paleo diet (18.7%), and raw food diet (17.9%), instead, were a matter of interest to almost the 18-39% of the respondents (**Figure 4** and **Supplementary Table 4**).

### 3.4 Patients’ information needs regarding the use of some foods and nutrients, in oncology

**Figure 5:**
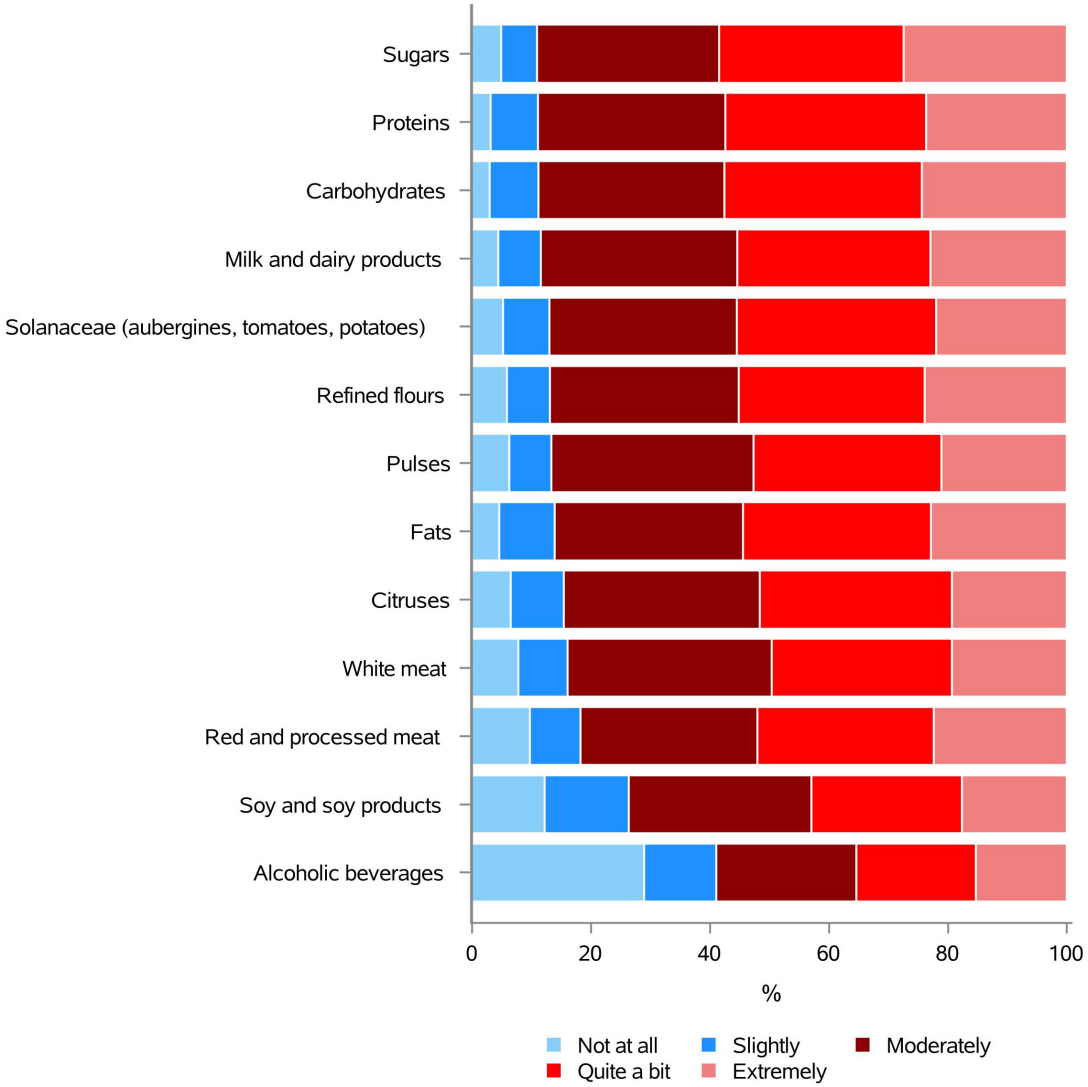
Information needs concerning the use of some foods and nutrients, in oncology.

Even if there is no clear scientific evidence indicating that the intake of specific foods/nutrients can ameliorate or worsening BC prognosis (6, 33, 37–45), many survivors express the urgent need to understand whether those can improve disease outcome (9, 10). Consistently, the results of our survey showed a very high need for information on these topics, indicating that 11 out of 13 foods/nutrients categories were of interest to our respondents with an average value of the 86.5% (**Figure 5** and **Supplementary Table 5**). Particularly, sugars (89.0%), proteins (88.8%), carbohydrates (88.7%), and milk and dairy products (88.4%) represented the top 4 choices. Only “soy and soy products” and, especially, alcoholic beverages were chosen by less than the 74% of the subjects, meaning the 73.6% and the 59.0% of the sample, respectively (**Figure 5** and **Supplementary Table 5**).

### 3.5 Patients’ information needs regarding the use of dietary supplements, in oncology

**Figure 6:**
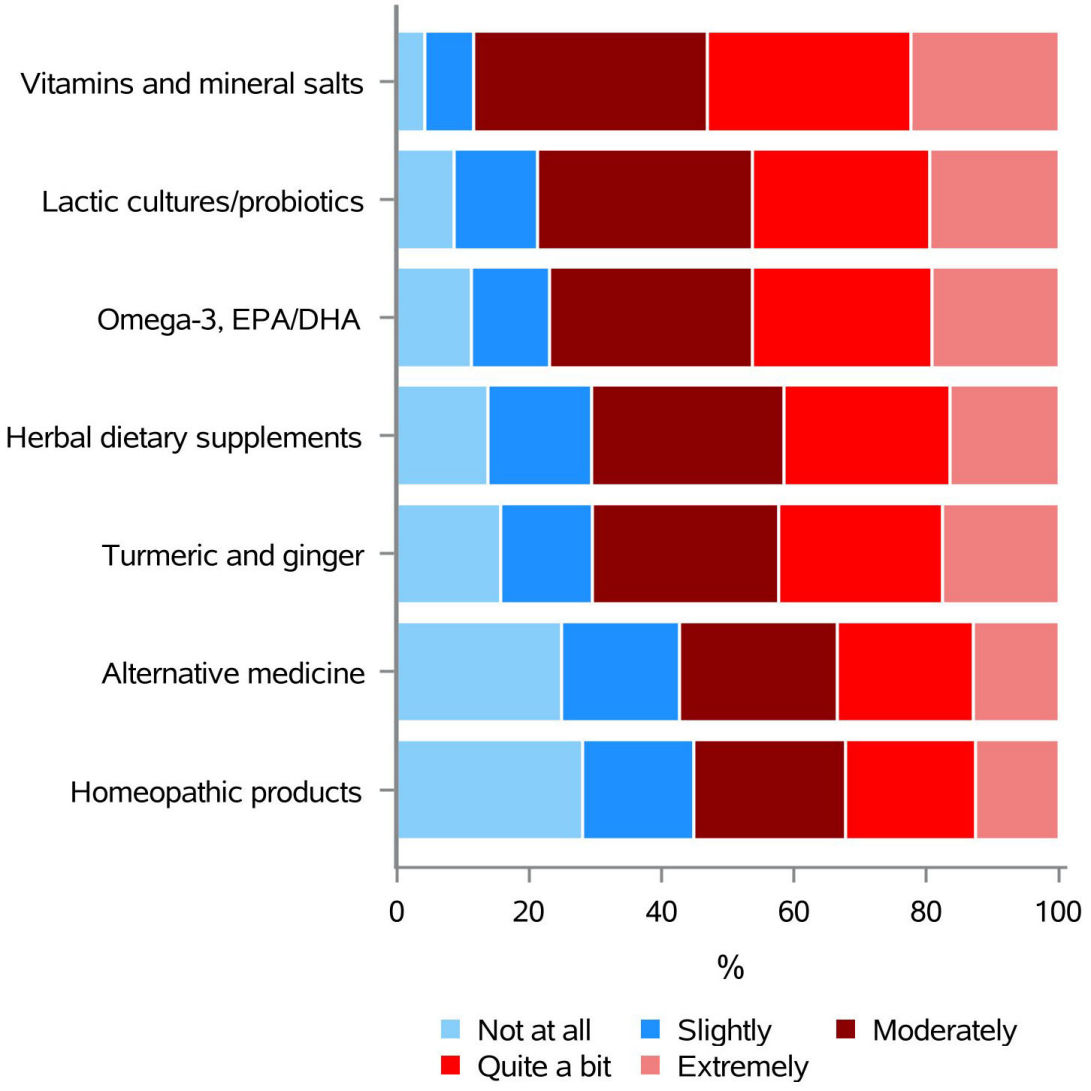
Information needs concerning the use of dietary supplements, in oncology.

Although dietary supplements are neither recommended to prevent breast cancer recurrence, nor to cure this malignancy, many BC patients take them without a physician’s guidance (9, 43). When asked about their need for information on the use of dietary supplements in oncology, respondents selected vitamins and mineral salts (88.4%) first, followed by lactic cultures/probiotics (78.7%), omega-3/EPA/DHA (76.9%), herbal dietary supplements (70.6%), turmeric and ginger (70.5%), alternative medicine (57.3%), and homeopathic products (55.2%) (**Figure 6** and **Supplementary Table 6**).

### 3.6 The first survey outcome: Instagram live broadcasts

Once intercepted and analyzed the Italian BC survivors’ nutrition information needs, as well as discovered where respondents would like to receive that information from, we first took advantage of the forthcoming Breast Cancer Awareness Month (October 2023) to organize Instagram live broadcasts, conducted by 3 dieticians/nutritionists, 1 oncologist and 1 psycho-oncologist, aimed at answering to “breast cancer and nutrition”-related questions. Notably, our respondents picked Facebook/Instagram as the fourth out of eleven communication channel options, just after healthcare professionals (referring physician/specialist doctor + nutritionist/dietitian), the Internet (institutional website), and books and/or traditional media (**Figure 2** and **Supplementary Table 2**).

Post engagements indicate the number of times people/followers have interacted with anything posted on a social media page. Engagement can take into account likes, comments, downloads, shares, direct messages and profile visits. Of course, it might be a good indicator to understand how many people are connecting with the information given on a social media post or live broadcast (63, 64). Social media engagement can be monitored in many ways and it has been considered good when the rate is between 1% and 5%, while an engagement rate below 1% is judged low (65).

The more likes, comments, shares, and downloads the higher the post engagement is; the higher the post engagement, the more likely it is that the published content will be appreciated by the audience and shared on other platforms.

To understand how successful our Instagram live broadcasts have been, we calculated the engagement rate for each of them, considering a time frame of 7 months (October 2023 - May 2024), using the following equation (65, 66).


(Likes+Comments+Saves)/Impression×100

Below, the engagement rates of each Instagram live broadcasts performed (**Supplementary Figure 1**).

A. Instagram live broadcast 1 (October 4, 2023): “Nutrition and breast cancer, what would you like to know? Diets”.
  (246 + 12 + 61)/8509 x 100 = 3.75%
B. Instagram live broadcast 2 (October 11, 2023): “Nutrition and breast cancer, what would you like to know? Foods and nutrients”.
  (302 + 24 + 83)/12443 x 100 = 3.3%
C. Instagram live broadcast 3 (October 20, 2023): “Nutrition and breast cancer, what would you like to know? Dietary supplements”.
  (205 + 11 + 51)/9536 x 100 = 2.8%
D. Instagram live broadcast 4 (October 25, 2023): “Nutrition and breast cancer, what would you like to know? Intolerance of uncertainty”.
  (77 + 0 + 14)/3818 x 100 = 2.4%
  The average overall engagement rate was 3.1%.

## 4 Discussion

In this work, we first intercepted Italian BC survivors’ nutrition information needs and current sources of advice, as well as ascertained where they would like to find information, in the future. Following that, we were able to address their information requirement with specific interventions aimed at giving patients reliable nutrition messages to follow, while counteracting deceiving and inaccurate communications. Another paper focusing on psycho-oncology themes, will report on whether and how anxiety status (Part 6 - question 19) affected the type and amount of information sought by our respondents.

Breast cancer survivors are known to be frequently concerned about diet and nutrition, thus they often express the need to obtain “cancer and nutrition”-related information in order to relieve side effects of cancer therapies, cure the disease or avoid cancer recurrence (8–11, 13). Moreover, studies have reported that, after a BC diagnosis, 30-60% of survivors commonly make nutritional changes, which sometimes are consistent with the guidelines for cancer prevention and the recommendations for a healthy diet during the follow up (9, 67–69). Even though we also have previously observed that some BC patients increased their consumption of vegetables, pulses, nuts, fruits, wholemeal bread/pasta, grains and fish, while decreasing red and processed meat, refined bread/pasta, baked good and animal fat intake, only less than 50% of them implemented those modifications (9). In contrast, we and other showed that 40-50% of BC patients reported having consumed dietary supplements after diagnosis (9, 10, 67–70). Besides, nutrition recommendations were recurrently found out from sources other than the healthcare professionals, such as search engines or social media, and dietetic changes pursued without informing the physician (9, 10, 17, 18). Even though social media can be a source of misinformation (24, 26, 28, 71–74), we cannot ignore the fact that they surely represent communication tools patients, their family and caregiver access to seek for information, comfort, or to talk about their experience. Also, both the Internet and social networks, might have the potential to give assistance and deliver the correct messages to cancer patients (49–58).

This knowledge helps us to structure the online survey “Nutrition and breast cancer, what would you like to know?”, where we identified and selected topics and questions covering many themes and information sources notably relevant for respondents the questionnaire was addressed to.

Previous papers showed patients often refer to the web as primary information source, finding it difficult to seek nutritional advice from healthcare professionals (9, 17, 18); in agreement with this, a significant proportion of our respondents declared to use the Internet, Facebook and Instagram as information sources. However, they preferentially chose institutional websites and, in addition, “referring physician/specialist doctor” and “nutritionist/dietitian” were selected as second and third option, respectively. This could either indicate subjects were incline to emphasize more “socially desirable” choices, or that sending our survey mainly through institutional websites and social networks might have selected a group of already quite well-informed BC patients. However, consistently with previous studies (14, 15, 18), we also found that age affects respondents’ source choice. In fact, while almost 60% of patients under 40 declared having used Facebook and Instagram “sometimes, often or always”, the same percentage of over 60 stated to access those social media only “never or rarely”. Moreover, 51.6% of the latter, when looking for information on nutrition and cancer, utilized books and/or traditional media, versus 32.3% of the under 40. Greater Internet and social network familiarity among younger respondents may explain those results.

As already showed in previous publications, despite survivors find difficult to obtain information from their physician, they would rather have access to many pieces of evidence through healthcare providers. Moreover, when this interaction occurs, it appears to be quite effective in addressing patient needs and concerns (75, 76). Interestingly, our work also found BC patients would strongly prefer to obtain information from the healthcare providers, choosing the referring physician/specialist doctor as first and nutritionist/dietitian as second option. The institutional websites were picked as third, followed by books and/or traditional media, clearly indicating a major need to receive evidence-based high-quality data. Besides, Facebook and Instagram were chosen as fifth option, just before the non-institutional websites. Again, social media were mainly selected by under 40 subjects, while less than 45% of those over 60 would use them. Podcast, in particular, emerged as an information resource almost the 50% of respondents under 40 would choose, whereas only the half of over 60 would pick it. Even if our respondents quite clearly indicated “institutional-like channel” as preferred sources to have information from, still social networks (especially Facebook and Instagram) and the Internet (both institutional and non-institutional websites), appear to be among the top ranked choices. These data, along with those related to the primary source of information consulted, are consistent with previous publications indicating that cancer patients use the Internet and social media to discuss their health with other survivors, seek comfort, and to search for various types of information (50, 53, 56–58). In addition, in many other cases where the doctor-patient relationship is not established, consultation with a health care professional is rare, and as a result, cancer patients rely on non-medical sources, including the Internet and social media, to understand their disease and/or even make decisions about it (77). In some instances, it has been observed that individuals are more inclined to engage with online misinformation than with factual information (24). This phenomenon can be attributed, at least in part, to the strong novelty character of fake news, which can lead some individuals to be unable to distinguish between true and false content (78). However, many papers have shown that, when correctly used, web-based information delivery could be a useful resource for BC survivors (49–58). Thus, we decided to respond to patients’ information needs by implementing two interventions: reply *via* Instagram live broadcasts, provided by healthcare professionals, to patients’ “cancer and nutrition”-related questions, and organize a healthcare providers dedicated-workshop - focused on nutrition and BC - to enable those professionals to answer patients with appropriate guidance, while hampering inaccurate and false messages. Both activities were based on respondents’ given answers about their need of information on: (i) nutrition and management of cancer treatment side effects; (ii) specific diets; (iii) foods and nutrients; and (iv) dietary supplements.

There is growing evidence that people diagnosed with BC often express a desire for nutritional information related to cancer treatment and its site effects (62). In line with this, most of our respondents were looking for information on osteoporosis, followed by weight gain, notably two topics of particular concern to this type of patient (6). In fact, many cancer therapies have the potential to induce early menopause and premature osteopenia, increasing the likelihood of osteoporosis and related complications, such as fractures (79). Weight gain is also an issue often associated with certain cancer treatments (anti-hormonal therapy, cortisone use, etc.), and can worry patients to the point of questioning adherence to therapy itself, resulting in an increased risk of future recurrences (5). Our results showed that BC patients still have a strong need for information on these specific topics, it is therefore of paramount importance to ensure that they are kept informed with simple, yet reliable and reassuring information that might promote adherence to cancer treatment.

It is not uncommon for BC patients to express concern about the most appropriate diet to follow after diagnosis. Indeed, many of them report a desire for information on this topic, with the aim of improving their health (9). In agreement with this, our respondents demonstrated a notable interest in various dietary regimens, including those that have been the subject of considerable debate in the field of oncology, such as fasting and dietary restrictions (80). Nevertheless, the diet most frequently selected by the majority of our sample was the Mediterranean diet. The observed outcome may be attributed to the fact that the respondents were Italian, and might have been exposed to information about this dietary approach in the past. This may have led to an interest in further learning or seeking clarification on a topic they were already familiar with, in the context of their disease. It would be beneficial to disseminate knowledge about the traditional Mediterranean model to patients, as this could prove to be a valuable and important resource. Indeed, the recommendations of the major international institutions on cancer prevention and management are in accordance with the characteristics of this dietary regimen (33, 36–40, 46–48).

Even though there is no obvious scientific evidence that specific foods or nutrients can improve or worsen BC prognosis (6, 33, 37–45), a significant proportion of survivors express a desire to ascertain whether these factors can influence disease outcome (9, 10). In accordance with the aforementioned findings, 11 out of 13 food and nutrient categories of our survey were of interest to our respondents, with an average value exceeding 86%. A number of those have previously been recognized as sources of fake news, especially on social networks. Sugar, in particular, generated the most interest (89%). Indeed, many patients wrongly believe there is a direct link between sugar consumption and the development of BC. It is therefore important to educate them about the evidence from the major international guidelines that prolonged sugary diets may lead to weight gain, and being overweight is associated with an increased risk of BC (6, 33, 36, 37, 39). Surprisingly, alcoholic beverages were identified as the element of the questionnaire that elicited the least interest. This is particularly worrying, given that alcohol consumption is a very well known risk factor for developing BC, both pre- and post-menopause (33, 37). This finding indicates that, regrettably, the increased cancer risk associated with alcohol consumption remains largely unacknowledged in the mass media, the internet, social media platforms, and, on some occasions, even by medical professionals themselves. This example further illustrates the importance of investigations, such as the one we conducted to determine the knowledge and need for information of a vulnerable target group.

The use of nutritional supplements for prevention, recurrence, or the treatment of BC is not recommended. Nevertheless, a considerable number of breast cancer patients do so without consulting a healthcare professional (9, 43). As could have been expected, the need for information on these subjects was also found to be high, with vitamins and mineral salts being selected by more than 88% of our respondents. This evidence indicates that it would be advantageous for healthcare professionals to inquire regularly of patients regarding their knowledge and usage of dietary supplements, while advising caution given the lack of evidence indicating their beneficial effects and the potential for increased risks, including cancer recurrence and harmful interactions with treatments (6, 33, 41–43, 45). Moreover, oncologists and other healthcare practitioners should be willing to answer questions regarding this topic and be prepared to provide guidance on the appropriate use of dietary supplements.

Once the Italian BC survivors’ nutritional information needs had been identified and their preferences for receiving this information established, the subsequent step was to capitalize on the upcoming Breast Cancer Awareness Month (October 2023) by organizing Instagram live broadcasts that would address questions related to the topic of “breast cancer and nutrition”. As other studies have indicated, the participation of healthcare professionals on social media offers a unique opportunity for direct engagement with patients. This approach can help provide information and facilitate real-time discussion about diagnosis and treatment experiences for those dealing with cancer (52). A live Instagram broadcast was also devoted to the effects of cancer on individuals’ lives, with the aim of discussing and examining the psychological impact of BC on patients. The level of engagement on social media has been regarded as satisfactory when it reaches a rate between 1% and 5% (65). The mean percentage of total audience engagement with our Instagram lives, within a period of 7 months, was determined to be 3.1%. This indicates that our broadcasts were successfully viewed and potentially shared on other online platforms, which may have resulted in the wider dissemination of correct information on the topic “nutrition and breast cancer”. This is another exemplary illustration of the potential for social networks to be utilized by health practitioners to disseminate clear, simple but scientifically accurate information to patients.

To this end, physicians must undergo training and remain up to date with the latest scientific evidence. In addition, they are required to possess the ability to communicate effectively and simply, in order to facilitate the transfer of knowledge. In light of the aforementioned factors, as well as the patients’ strong desire to obtain satisfactory responses from their referring physician/specialist doctor and nutritionist/dietitian, we have decided to create an accredited course for healthcare professionals. This course is designed to provide a comprehensive training in the topic of “nutrition and lifestyle before, during and after a breast cancer diagnosis.” It also aims to develop participants’ communication skills, enabling them to effectively convey the latest scientific information in a simple and practical manner, suitable for both patients and their caregivers.

By analyzing the data collected in our questionnaire, it will become possible to implement further actions in the future. One such action could be to create or enforce documents dedicated to the topics that have aroused the greatest level of interest, in relation to the subject of “nutrition and breast cancer”. These documents could then be disseminated on institutional websites. Moreover, the publication of a book on those topics would serve as an excellent vehicle for reaching a broader audience, including those who prefer to stay up to date using more traditional media.

It is our contention that adapting the survey “Nutrition and breast cancer, what would you like to know?” to analyze the information needs of patients diagnosed with other types of tumors would be of significant value. Additionally, it would be beneficial to be able to reach a larger and more heterogeneous group of subjects. For example, taking into account the type/grade of cancer and the treatment received may be useful to help better understand the information needs of specific groups of survivors. The involvement of local, regional, municipal organizations or associations might represent an efficacious approach to reaching specific target audiences, including older individuals or those engaged in sports activities. This could be achieved by organizing workshops, seminars, or webinars, which may be tailored to align with the unique characteristics and needs of each identified community on a case-by-case basis.

The present work has some limitations. Due to the self-reported nature of the survey, patients may have been inclined to provide, in some cases, more socially desirable, inaccurate, or biased responses, either intentionally (to conceal information or appear different than they are) or unintentionally (due to misunderstanding or inaccuracy). Also, respondents may have encountered difficulties in interpreting or fully understanding questions, leading to inaccurate or incorrect answers. Finally, since our questionnaire was primarily distributed through institutional channels, hospitals, and social networks of health professionals, it probably resulted in selecting a group of respondents who were already quite knowledgeable about the issues in question and accustomed to using more scientifically recognized channels.

However, two major strengths must be considered. To the best of our knowledge, this is the first study to investigate the need for information on cancer and nutrition in a group of Italian patients diagnosed with BC. Furthermore, the analysis was carried out using a previously validated, easy-to-use and understandable tool (Microsoft Forms) (81, 82) that can also be adapted for administration to patients with other forms of tumors.

## 5 Conclusion

Our study showed that, despite the existence of many options and various forms of access to health information, our group of Italian BC respondents would primarily desire to receive such information from their referring physician, specialist, nutritionist, or dietitian. This is further proof that healthcare providers must identify patients’ information needs (including through social media) and develop, implement, and disseminate reliable, *ad hoc* dietary and lifestyle recommendations using patients’ preferred communication channels. Therefore, it is imperative that healthcare professionals be provided with education and training on these topics so that they can play a greater role in delivering appropriate dietary advice, counseling, and education to patients and their caregivers.

## Data Availability

The raw data supporting the conclusions of this article will be made available by the authors, without undue reservation.
