# Supplementary material for: Results of the Italian cross-sectional web-based survey “Nutrition and breast cancer, what would you like to know?” An attempt to collect and respond to patients’ information needs, through social media
Source: Front Oncol. 2024 Sep 25;14:1436610. doi: 10.3389/fonc.2024.1436610 (PMC11461502; doi:10.3389/fonc.2024.1436610)
Supplement: Supplementary file 1 [file DataSheet1.docx]

Supplementary Material

# Supplementary Data

**SUPPLEMENTARY FILE 1**. The online questionnaire “Nutrition and breast cancer, what would you like to know?” consisting of 6 main sections for a total of 19 questions.

**Survey: “Nutrition and breast cancer, what would you like to know?”**

Dear Respondent,

the survey “Nutrition and breast cancer, what would you like to know?” was conceived, designed, reviewed and approved by the European Institute of Oncology (IEO). The purpose of this questionnaire is to understand your information needs (about the management of cancer treatment side effects, nutrients and dietary supplements), identify your current sources of advice, as well as ascertaining where you would rather find reliable information in the future.

The questionnaire is simple to complete and will only take a few minutes of your time.

Data are collected in a way that does not directly identify anyone. Only the answers provided to the questionnaire, after acknowledging and comprehending our terms and conditions of use, will be collected and analyzed by IEO (Data Controller of the personal data), under the European Union General Data Protection Regulation (EU 2016/679).

If you wish, you can contact the Data Protection Officer of IEO for any request regarding the protection of your personal data and the exercise of your rights, by writing to the following email address: [privacy@ieo.it](mailto:privacy@ieo.it) or [direzione.sanitaria@ieo.it](mailto:direzione.sanitaria@ieo.it).

**The questionnaire is structured into six main sections.**

**Part 1**

Personal details and information sources.

**Part 2**

Management of cancer treatment side effects.

**Part 3**

Specific diets.

**Part 4**

Foods and nutrients.

**Part 5**

Dietary supplements.

**Part 6**

Uncertainty management.

**How to fill in the form**

Answer each question in the questionnaire by ticking the relevant answer. If you are uncertain, choose the answer that fits best.

Questions marked with a red asterisk (*) are required. To correctly complete and send the questionnaire, you must always select only one answer per row (questions 8, 10, 12, 13, 14, 15, 16 and 19).

Bear in mind that this questionnaire is not an evaluation and there are no right or wrong answers.

***If you wish to fill in the questionnaire using your phone, turn it sideways so that you can better see answer options!***

Thank you for taking the time to complete this questionnaire for us.

**Part 1**

**1. I voluntarily agree to complete the questionnaire, without providing any data allowing my direct identification, and I am aware that the information here provided will be used for scientific dissemination and research purposes only. ***

〇 Please, tick the box to show your consent.

**2. How did you hear about this questionnaire? ***

〇 Smartfood and/or IEO communication channels (social networks, newsletters and websites).

〇 Flyer on hospital wall.

〇 Word of mouth.

**3. How old are you? ***

Please, select your age from the drop-down menu (18-90 years)

**4. Sex ***

〇 F

〇 M

〇 I’d rather not say

**5. Select your region from the drop-down menu ***

Abruzzo

Basilicata

Calabria

Campania

Emilia-Romagna

Friuli-Venezia Giulia

Lazio

Liguria

Lombardy

Brands

Molise

Piedmont

Puglia

Sardinia

Sicily

Tuscany

Trentino-Alto Adige

Umbria

Aosta Valley

Veneto

**6. What is the highest degree or level of school you have completed? If currently enrolled, select the highest degree received ***

〇 No schooling completed.

〇 Primary school.

〇 Junior high school.

〇 High school.

〇 Master’s degree.

〇 Postgraduate degree.

**7. Choose the definition that suits you best ***

〇 Person diagnosed with breast cancer.

〇 Relative, parent, spouse or partner of a person diagnosed with breast cancer.

〇 Friend of a person diagnosed with breast cancer.

〇 Caregiver of a person diagnosed with breast cancer.

〇 Healthcare professional (physician, oncologist, nurse, dietitian, nutritionist, pharmacist etc.)

〇 Other.

**8. Currently, where do you get information about “quality of life and nutrition in oncology” from?**

**For each category choose only one option among: Never, Rarely, Sometimes, Often and Always.**

***Note! Scroll down until you see all the options.* ***

|  | **Never** | **Rarely** | **Sometimes** | **Often** | **Always** |
| --- | --- | --- | --- | --- | --- |
| The Internet (non-institutional websites - blogs, forums, personal web pages, amateur websites, online magazines etc.) |  |  |  |  |  |
| The Internet (institutional websites - owned and operated by hospitals, universities, colleges, seminaries, institutes of technology, state agencies etc.) |  |  |  |  |  |
| Facebook and Instagram |  |  |  |  |  |
| YouTube |  |  |  |  |  |
| TikTok |  |  |  |  |  |
| Whatsapp groups |  |  |  |  |  |
| Telegram groups |  |  |  |  |  |
| Books and/or traditional media (television, radio, newspapers, magazines etc.) |  |  |  |  |  |
| Podcast |  |  |  |  |  |
| Referring physician/specialist doctor |  |  |  |  |  |
| Nutritionist/dietitian |  |  |  |  |  |
| Other* |  |  |  |  |  |

**9. *: If you selected “Other*” (Rarely, Sometimes, Often or Always), in question 8, please specify.**

Not required question.

**10. In the future, where would you like to have information about “quality of life and nutrition in oncology” from?**

**For each category choose only one option among: Never, Rarely, Sometimes, Often and Always.**

***Note! Scroll down until you see all the options.* ***

|  | **Never** | **Rarely** | **Sometimes** | **Often** | **Always** |
| --- | --- | --- | --- | --- | --- |
| The Internet (non-institutional websites - blogs, forums, personal web pages, amateur websites, online magazines etc.) |  |  |  |  |  |
| The Internet (institutional websites - owned and operated by hospitals, universities, colleges, seminaries, institutes of technology, state agencies etc.) |  |  |  |  |  |
| Facebook and Instagram |  |  |  |  |  |
| YouTube |  |  |  |  |  |
| TikTok |  |  |  |  |  |
| Whatsapp groups |  |  |  |  |  |
| Telegram groups |  |  |  |  |  |
| Books and/or traditional media (television, radio, newspapers, magazines etc.) |  |  |  |  |  |
| Podcast |  |  |  |  |  |
| Referring physician/specialist doctor |  |  |  |  |  |
| Nutritionist/dietitian |  |  |  |  |  |
| Other* |  |  |  |  |  |

**11. *: If you selected “Other*” (Rarely, Sometimes, Often or Always), in question 10, please specify.**

Not required question.

**Part 2**

**12. How much do you need to know about the effects of specific diets, foods, nutrients and dietary supplements in managing side effects of the following cancer treatments?**

**For each category choose only one option among: Not at all, Slightly, Moderately, Quite a bit, Extremely.**

***Note! Scroll down until you see all the options.* ***

|  | **Not at all** | **Slightly** | **Moderately** | **Quite a bit** | **Extremely** |
| --- | --- | --- | --- | --- | --- |
| Chemotherapy |  |  |  |  |  |
| Radiotherapy |  |  |  |  |  |
| Hormone therapy |  |  |  |  |  |
| Hormone therapy + Ribociclib/Abemaciclib/Palbociclib |  |  |  |  |  |
| Immunotherapy |  |  |  |  |  |
| Anti-HER2 therapies (Trastuzumab, TDM-1, Pertuzumab etc.) |  |  |  |  |  |
| Olaparib or Talazoparib |  |  |  |  |  |
| Drugs for the treatment of bone metastases |  |  |  |  |  |

**13. How much do you need to know about the effects of specific diets, foods, nutrients and dietary supplements in managing the following cancer treatment side effects?**

**For each category choose only one option among: Not at all, Slightly, Moderately, Quite a bit, Extremely.**

***Note! Scroll down until you see all the options.* ***

|  | **Not at all** | **Slightly** | **Moderately** | **Quite a bit** | **Extremely** |
| --- | --- | --- | --- | --- | --- |
| Changes in taste |  |  |  |  |  |
| Changes in lipid profile (e.g. increased cholesterol) |  |  |  |  |  |
| Weight gain |  |  |  |  |  |
| Diarrhea |  |  |  |  |  |
| Dehydration |  |  |  |  |  |
| Fatigue |  |  |  |  |  |
| Gastritis |  |  |  |  |  |
| Abdominal swelling |  |  |  |  |  |
| Subcutaneous swelling/edema |  |  |  |  |  |
| Nausea and vomiting |  |  |  |  |  |
| Osteoporosis |  |  |  |  |  |
| Loss of appetite |  |  |  |  |  |
| Cognitive dysfunction (e.g. diminished concentration or attention, memory loss) |  |  |  |  |  |
| Dry mouth |  |  |  |  |  |
| Constipation |  |  |  |  |  |
| Stomatitis |  |  |  |  |  |

**Part 3**

**14. How much do you need to know about the use of the following diets in oncology?**

**For each category choose only one option among: Not at all, Slightly, Moderately, Quite a bit, Extremely.**

***Note! Scroll down until you see all the options.* ***

|  | **Not at all** | **Slightly** | **Moderately** | **Quite a bit** | **Extremely** |
| --- | --- | --- | --- | --- | --- |
| Mediterranean diet |  |  |  |  |  |
| Vegetarian diet |  |  |  |  |  |
| Vegan diet |  |  |  |  |  |
| Macrobiotic diet |  |  |  |  |  |
| High-protein diet |  |  |  |  |  |
| High-calorie diet |  |  |  |  |  |
| Ketogenic diet |  |  |  |  |  |
| Blood-type diet |  |  |  |  |  |
| Acid-alkaline diet |  |  |  |  |  |
| Low-glycemic index diet |  |  |  |  |  |
| Paleo diet |  |  |  |  |  |
| Raw food diet |  |  |  |  |  |
| Detox diet/Cleansing diet |  |  |  |  |  |
| Fasting (total fasting, alternate day fasting, periodic fasting, fasting-mimicking diet, intermittent fasting 5/2 days, intermittent fasting 16/8 hours etc.) |  |  |  |  |  |
| Dietary restrictions (low-calorie diet, protein-restricted diet, carbohydrate-restricted diet etc.) |  |  |  |  |  |

**Part 4**

**15. How much do you need to know about the use of the following foods and nutrients in oncology?**

**For each category choose only one option among: Not at all, Slightly, Moderately, Quite a bit, Extremely.**

***Note! Scroll down until you see all the options.* ***

|  | **Not at all** | **Slightly** | **Moderately** | **Quite a bit** | **Extremely** |
| --- | --- | --- | --- | --- | --- |
| Soy and soy products |  |  |  |  |  |
| Milk and dairy products |  |  |  |  |  |
| Sugars |  |  |  |  |  |
| Refined flours |  |  |  |  |  |
| Red and processed meat |  |  |  |  |  |
| White meat |  |  |  |  |  |
| Alcoholic beverages |  |  |  |  |  |
| Pulses |  |  |  |  |  |
| Citruses |  |  |  |  |  |
| Solanaceae (aubergines, tomatoes, potatoes) |  |  |  |  |  |
| Carbohydrates |  |  |  |  |  |
| Fats |  |  |  |  |  |
| Proteins |  |  |  |  |  |

**Part 5**

**16. How much do you need to know about the use of the following dietary supplements in oncology?**

**For each category choose only one option among: Not at all, Slightly, Moderately, Quite a bit, Extremely.**

***Note! Scroll down until you see all the options.* ***

|  | **Not at all** | **Slightly** | **Moderately** | **Quite a bit** | **Extremely** |
| --- | --- | --- | --- | --- | --- |
| Vitamins and mineral salts |  |  |  |  |  |
| Alternative medicine (Bach flowers, traditional Chinese medicine) |  |  |  |  |  |
| Herbal dietary supplements (herbal teas, infusions, decoctions, essential oils, mother tinctures etc.) |  |  |  |  |  |
| Homeopathic products |  |  |  |  |  |
| Lactic cultures/probiotics |  |  |  |  |  |
| Omega-3, EPA/DHA |  |  |  |  |  |
| Turmeric and ginger |  |  |  |  |  |

**17. Do you need to learn more about “nutrition and breast cancer”? ***

〇 YES

〇 NO

If you answered YES to question 17, please proceed to required question 18.

If you answered NO, please proceed to required question 19.

**18. Please, specify which information you wish to receive. ***

**Part 6**

**19. You will find below a series of statements which describe how people may react to the uncertainties of life. Please use the scale below to describe to what extent each item is characteristic of you. Please circle a number (1 to 5) that describes you best. The scale is anchored at 1 = not at all characteristic of me to 5 = entirely characteristic of me. Scores of 3 indicate “somewhat characteristic of me”. ***

|  | **1** | **2** | **3** | **4** | **5** |
| --- | --- | --- | --- | --- | --- |
| Unforeseen events upset me greatly |  |  |  |  |  |
| It frustrates me not having all the information I need |  |  |  |  |  |
| One should always look ahead so as to avoid surprises |  |  |  |  |  |
| A small, unforeseen event can spoil everything, even with the best of planning |  |  |  |  |  |
| I always want to know what the future has in store for me |  |  |  |  |  |
| I can’t stand being taken by surprise |  |  |  |  |  |
| I should be able to organize everything in advance |  |  |  |  |  |
| Uncertainty keeps me from living a full life |  |  |  |  |  |
| When it’s time to act, uncertainty paralyses me |  |  |  |  |  |
| When I am uncertain, I can’t function very well |  |  |  |  |  |
| The smallest doubt can stop me from acting |  |  |  |  |  |
| I must get away from all uncertain situations |  |  |  |  |  |

**Supplementary Table 1**. Information sources currently consulted about “quality of life and nutrition in oncology”, overall and according to age class (<40, 40-49, 50-59, ≥60 years).

(N = number of respondents, % = percentage of respondents).

|  | **Age class (years)** | | | | | | | | **Overall**  **(N=1159)** | |
| --- | --- | --- | --- | --- | --- | --- | --- | --- | --- | --- |
|  | **<40**  **(N=161)** | | **40-49**  **(N=426)** | | **50-59**  **(N=417)** | | **≥60**  **(N=155)** | |  |  |
|  | **N** | **%** | **N** | **%** | **N** | **%** | **N** | **%** | **N** | **%** |
| **The Internet (non-institutional websites)** |  |  |  |  |  |  |  |  |  |  |
| Never | 28 | 17.4 | 66 | 15.5 | 94 | 22.5 | 44 | 28.4 | 232 | 20.0 |
| Rarely | 51 | 31.7 | 125 | 29.3 | 134 | 32.1 | 52 | 33.5 | 362 | 31.2 |
| Sometimes | 41 | 25.5 | 140 | 32.9 | 109 | 26.1 | 39 | 25.2 | 329 | 28.4 |
| Often | 27 | 16.8 | 68 | 16.0 | 65 | 15.6 | 17 | 11.0 | 177 | 15.3 |
| Always | 14 | 8.7 | 27 | 6.3 | 15 | 3.6 | 3 | 1.9 | 59 | 5.1 |
| **The Internet (institutional websites)** |  |  |  |  |  |  |  |  |  |  |
| Never | 3 | 1.9 | 17 | 4.0 | 23 | 5.5 | 15 | 9.7 | 58 | 5.0 |
| Rarely | 35 | 21.7 | 67 | 15.7 | 85 | 20.4 | 18 | 11.6 | 205 | 17.7 |
| Sometimes | 46 | 28.6 | 177 | 41.5 | 134 | 32.1 | 51 | 32.9 | 408 | 35.2 |
| Often | 45 | 28.0 | 108 | 25.4 | 123 | 29.5 | 50 | 32.3 | 326 | 28.1 |
| Always | 32 | 19.9 | 57 | 13.4 | 52 | 12.5 | 21 | 13.5 | 162 | 14.0 |
| **Facebook and Instagram** |  |  |  |  |  |  |  |  |  |  |
| Never | 25 | 15.5 | 77 | 18.1 | 104 | 24.9 | 52 | 33.5 | 258 | 22.3 |
| Rarely | 40 | 24.8 | 124 | 29.1 | 123 | 29.5 | 41 | 26.5 | 328 | 28.3 |
| Sometimes | 59 | 36.6 | 126 | 29.6 | 99 | 23.7 | 35 | 22.6 | 319 | 27.5 |
| Often | 24 | 14.9 | 69 | 16.2 | 68 | 16.3 | 20 | 12.9 | 181 | 15.6 |
| Always | 13 | 8.1 | 30 | 7.0 | 23 | 5.5 | 7 | 4.5 | 73 | 6.3 |
| **YouTube** |  |  |  |  |  |  |  |  |  |  |
| Never | 108 | 67.1 | 280 | 65.7 | 274 | 65.7 | 104 | 67.1 | 766 | 66.1 |
| Rarely | 33 | 20.5 | 108 | 25.4 | 93 | 22.3 | 31 | 20.0 | 265 | 22.9 |
| Sometimes | 15 | 9.3 | 23 | 5.4 | 40 | 9.6 | 14 | 9.0 | 92 | 7.9 |
| Often | 4 | 2.5 | 11 | 2.6 | 9 | 2.2 | 5 | 3.2 | 29 | 2.5 |
| Always | 1 | 0.6 | 4 | 0.9 | 1 | 0.2 | 1 | 0.6 | 7 | 0.6 |
| **TikTok** |  |  |  |  |  |  |  |  |  |  |
| Never | 142 | 88.2 | 384 | 90.1 | 369 | 88.5 | 142 | 91.6 | 1037 | 89.5 |
| Rarely | 12 | 7.5 | 28 | 6.6 | 33 | 7.9 | 8 | 5.2 | 81 | 7.0 |
| Sometimes | 7 | 4.3 | 12 | 2.8 | 10 | 2.4 | 4 | 2.6 | 33 | 2.8 |
| Often | 0 | - | 2 | 0.5 | 4 | 1.0 | 1 | 0.6 | 7 | 0.6 |
| Always | 0 | - | 0 | - | 1 | 0.2 | 0 | - | 1 | 0.1 |
| **Whatsapp groups** |  |  |  |  |  |  |  |  |  |  |
| Never | 138 | 85.7 | 337 | 79.1 | 312 | 74.8 | 113 | 72.9 | 900 | 77.7 |
| Rarely | 14 | 8.7 | 52 | 12.2 | 58 | 13.9 | 23 | 14.8 | 147 | 12.7 |
| Sometimes | 9 | 5.6 | 24 | 5.6 | 27 | 6.5 | 13 | 8.4 | 73 | 6.3 |
| Often | 0 | - | 11 | 2.6 | 18 | 4.3 | 6 | 3.9 | 35 | 3.0 |
| Always | 0 | - | 2 | 0.5 | 2 | 0.5 | 0 | - | 4 | 0.3 |
| **Telegram groups** |  |  |  |  |  |  |  |  |  |  |
| Never | 150 | 93.2 | 387 | 90.8 | 367 | 88.0 | 138 | 89.0 | 1042 | 89.9 |
| Rarely | 6 | 3.7 | 29 | 6.8 | 36 | 8.6 | 14 | 9.0 | 85 | 7.3 |
| Sometimes | 5 | 3.1 | 8 | 1.9 | 11 | 2.6 | 2 | 1.3 | 26 | 2.2 |
| Often | 0 | - | 2 | 0.5 | 2 | 0.5 | 1 | 0.6 | 5 | 0.4 |
| Always | 0 | - | 0 | - | 1 | 0.2 | 0 | - | 1 | 0.1 |
| **Books and/or traditional media** |  |  |  |  |  |  |  |  |  |  |
| Never | 55 | 34.2 | 66 | 15.5 | 65 | 15.6 | 22 | 14.2 | 208 | 17.9 |
| Rarely | 54 | 33.5 | 175 | 41.1 | 141 | 33.8 | 53 | 34.2 | 423 | 36.5 |
| Sometimes | 31 | 19.3 | 113 | 26.5 | 132 | 31.7 | 47 | 30.3 | 323 | 27.9 |
| Often | 15 | 9.3 | 52 | 12.2 | 58 | 13.9 | 25 | 16.1 | 150 | 12.9 |
| Always | 6 | 3.7 | 20 | 4.7 | 21 | 5.0 | 8 | 5.2 | 55 | 4.7 |
| **Podcast** |  |  |  |  |  |  |  |  |  |  |
| Never | 100 | 62.1 | 294 | 69.0 | 292 | 70.0 | 100 | 64.5 | 786 | 67.8 |
| Rarely | 31 | 19.3 | 63 | 14.8 | 73 | 17.5 | 28 | 18.1 | 195 | 16.8 |
| Sometimes | 21 | 13.0 | 44 | 10.3 | 39 | 9.4 | 19 | 12.3 | 123 | 10.6 |
| Often | 8 | 5.0 | 20 | 4.7 | 9 | 2.2 | 4 | 2.6 | 41 | 3.5 |
| Always | 1 | 0.6 | 5 | 1.2 | 4 | 1.0 | 4 | 2.6 | 14 | 1.2 |
| **Referring physician/specialist doctor** |  |  |  |  |  |  |  |  |  |  |
| Never | 15 | 9.3 | 31 | 7.3 | 26 | 6.2 | 12 | 7.7 | 84 | 7.2 |
| Rarely | 38 | 23.6 | 89 | 20.9 | 71 | 17.0 | 22 | 14.2 | 220 | 19.0 |
| Sometimes | 38 | 23.6 | 140 | 32.9 | 128 | 30.7 | 36 | 23.2 | 342 | 29.5 |
| Often | 30 | 18.6 | 69 | 16.2 | 86 | 20.6 | 42 | 27.1 | 227 | 19.6 |
| Always | 40 | 24.8 | 97 | 22.8 | 106 | 25.4 | 43 | 27.7 | 286 | 24.7 |
| **Nutritionist/dietitian** |  |  |  |  |  |  |  |  |  |  |
| Never | 34 | 21.1 | 94 | 22.1 | 105 | 25.2 | 34 | 21.9 | 267 | 23.0 |
| Rarely | 27 | 16.8 | 94 | 22.1 | 65 | 15.6 | 31 | 20.0 | 217 | 18.7 |
| Sometimes | 44 | 27.3 | 115 | 27.0 | 100 | 24.0 | 32 | 20.6 | 291 | 25.1 |
| Often | 21 | 13.0 | 60 | 14.1 | 74 | 17.7 | 30 | 19.4 | 185 | 16.0 |
| Always | 35 | 21.7 | 63 | 14.8 | 73 | 17.5 | 28 | 18.1 | 199 | 17.2 |

**Supplementary Table 2**. Preferred sources for receiving information, in the future, about “quality of life and nutrition in oncology”, overall and according to age class (<40, 40-49, 50-59, ≥60 years).

(N = number of respondents, % = percentage of respondents).

|  | **Age class (years)** | | | | | | | | **Overall**  **(N=1159)** | |
| --- | --- | --- | --- | --- | --- | --- | --- | --- | --- | --- |
|  | **<40**  **(N=161)** | | **40-49**  **(N=426)** | | **50-59**  **(N=417)** | | **≥60**  **(N=155)** | |  |  |
|  | **N** | **%** | **N** | **%** | **N** | **%** | **N** | **%** | **N** | **%** |
| **The Internet (non-institutional websites)** |  |  |  |  |  |  |  |  |  |  |
| Never | 52 | 32.3 | 105 | 24.6 | 131 | 31.4 | 57 | 36.8 | 345 | 29.8 |
| Rarely | 31 | 19.3 | 102 | 23.9 | 101 | 24.2 | 48 | 31.0 | 282 | 24.3 |
| Sometimes | 40 | 24.8 | 125 | 29.3 | 98 | 23.5 | 32 | 20.6 | 295 | 25.5 |
| Often | 22 | 13.7 | 55 | 12.9 | 60 | 14.4 | 12 | 7.7 | 149 | 12.9 |
| Always | 16 | 9.9 | 39 | 9.2 | 27 | 6.5 | 6 | 3.9 | 88 | 7.6 |
| **The Internet (institutional websites)** |  |  |  |  |  |  |  |  |  |  |
| Never | 6 | 3.7 | 17 | 4.0 | 25 | 6.0 | 8 | 5.2 | 56 | 4.8 |
| Rarely | 6 | 3.7 | 26 | 6.1 | 40 | 9.6 | 13 | 8.4 | 85 | 7.3 |
| Sometimes | 46 | 28.6 | 136 | 31.9 | 122 | 29.3 | 44 | 28.4 | 348 | 30.0 |
| Often | 38 | 23.6 | 104 | 24.4 | 100 | 24.0 | 40 | 25.8 | 282 | 24.3 |
| Always | 65 | 40.4 | 143 | 33.6 | 130 | 31.2 | 50 | 32.3 | 388 | 33.5 |
| **Facebook and Instagram** |  |  |  |  |  |  |  |  |  |  |
| Never | 34 | 21.1 | 98 | 23.0 | 124 | 29.7 | 50 | 32.3 | 306 | 26.4 |
| Rarely | 23 | 14.3 | 77 | 18.1 | 86 | 20.6 | 36 | 23.2 | 222 | 19.2 |
| Sometimes | 48 | 29.8 | 130 | 30.5 | 104 | 24.9 | 29 | 18.7 | 311 | 26.8 |
| Often | 33 | 20.5 | 72 | 16.9 | 77 | 18.5 | 30 | 19.4 | 212 | 18.3 |
| Always | 23 | 14.3 | 49 | 11.5 | 26 | 6.2 | 10 | 6.5 | 108 | 9.3 |
| **YouTube** |  |  |  |  |  |  |  |  |  |  |
| Never | 85 | 52.8 | 262 | 61.5 | 251 | 60.2 | 91 | 58.7 | 689 | 59.4 |
| Rarely | 20 | 12.4 | 64 | 15.0 | 75 | 18.0 | 27 | 17.4 | 186 | 16.0 |
| Sometimes | 32 | 19.9 | 63 | 14.8 | 62 | 14.9 | 23 | 14.8 | 180 | 15.5 |
| Often | 16 | 9.9 | 21 | 4.9 | 22 | 5.3 | 11 | 7.1 | 70 | 6.0 |
| Always | 8 | 5.0 | 16 | 3.8 | 7 | 1.7 | 3 | 1.9 | 34 | 2.9 |
| **TikTok** |  |  |  |  |  |  |  |  |  |  |
| Never | 127 | 78.9 | 355 | 83.3 | 340 | 81.5 | 129 | 83.2 | 951 | 82.1 |
| Rarely | 16 | 9.9 | 33 | 7.7 | 46 | 11.0 | 17 | 11.0 | 112 | 9.7 |
| Sometimes | 11 | 6.8 | 26 | 6.1 | 18 | 4.3 | 8 | 5.2 | 63 | 5.4 |
| Often | 5 | 3.1 | 5 | 1.2 | 7 | 1.7 | 1 | 0.6 | 18 | 1.6 |
| Always | 2 | 1.2 | 7 | 1.6 | 6 | 1.4 | 0 | - | 15 | 1.3 |
| **Whatsapp groups** |  |  |  |  |  |  |  |  |  |  |
| Never | 111 | 68.9 | 277 | 65.0 | 272 | 65.2 | 98 | 63.2 | 758 | 65.4 |
| Rarely | 19 | 11.8 | 50 | 11.7 | 68 | 16.3 | 25 | 16.1 | 162 | 14.0 |
| Sometimes | 17 | 10.6 | 63 | 14.8 | 42 | 10.1 | 20 | 12.9 | 142 | 12.3 |
| Often | 11 | 6.8 | 19 | 4.5 | 23 | 5.5 | 9 | 5.8 | 62 | 5.3 |
| Always | 3 | 1.9 | 17 | 4.0 | 12 | 2.9 | 3 | 1.9 | 35 | 3.0 |
| **Telegram groups** |  |  |  |  |  |  |  |  |  |  |
| Never | 126 | 78.3 | 326 | 76.5 | 324 | 77.7 | 127 | 81.9 | 903 | 77.9 |
| Rarely | 12 | 7.5 | 38 | 8.9 | 50 | 12.0 | 16 | 10.3 | 116 | 10.0 |
| Sometimes | 13 | 8.1 | 45 | 10.6 | 25 | 6.0 | 10 | 6.5 | 93 | 8.0 |
| Often | 7 | 4.3 | 6 | 1.4 | 12 | 2.9 | 1 | 0.6 | 26 | 2.2 |
| Always | 3 | 1.9 | 11 | 2.6 | 6 | 1.4 | 1 | 0.6 | 21 | 1.8 |
| **Books and/or traditional media** |  |  |  |  |  |  |  |  |  |  |
| Never | 29 | 18.0 | 56 | 13.1 | 61 | 14.6 | 18 | 11.6 | 164 | 14.2 |
| Rarely | 22 | 13.7 | 73 | 17.1 | 76 | 18.2 | 29 | 18.7 | 200 | 17.3 |
| Sometimes | 34 | 21.1 | 128 | 30.0 | 116 | 27.8 | 37 | 23.9 | 315 | 27.2 |
| Often | 32 | 19.9 | 90 | 21.1 | 109 | 26.1 | 48 | 31.0 | 279 | 24.1 |
| Always | 44 | 27.3 | 79 | 18.5 | 55 | 13.2 | 23 | 14.8 | 201 | 17.3 |
| **Podcast** |  |  |  |  |  |  |  |  |  |  |
| Never | 65 | 40.4 | 214 | 50.2 | 224 | 53.7 | 90 | 58.1 | 593 | 51.2 |
| Rarely | 16 | 9.9 | 57 | 13.4 | 66 | 15.8 | 25 | 16.1 | 164 | 14.2 |
| Sometimes | 30 | 18.6 | 80 | 18.8 | 65 | 15.6 | 22 | 14.2 | 197 | 17.0 |
| Often | 25 | 15.5 | 41 | 9.6 | 40 | 9.6 | 12 | 7.7 | 118 | 10.2 |
| Always | 25 | 15.5 | 34 | 8.0 | 22 | 5.3 | 6 | 3.9 | 87 | 7.5 |
| **Referring physician/specialist doctor** |  |  |  |  |  |  |  |  |  |  |
| Never | 0 | - | 7 | 1.6 | 11 | 2.6 | 2 | 1.3 | 20 | 1.7 |
| Rarely | 2 | 1.2 | 9 | 2.1 | 11 | 2.6 | 6 | 3.9 | 28 | 2.4 |
| Sometimes | 38 | 23.6 | 108 | 25.4 | 80 | 19.2 | 30 | 19.4 | 256 | 22.1 |
| Often | 20 | 12.4 | 68 | 16.0 | 85 | 20.4 | 35 | 22.6 | 208 | 17.9 |
| Always | 101 | 62.7 | 234 | 54.9 | 230 | 55.2 | 82 | 52.9 | 647 | 55.8 |
| **Nutritionist/dietitian** |  |  |  |  |  |  |  |  |  |  |
| Never | 4 | 2.5 | 22 | 5.2 | 30 | 7.2 | 10 | 6.5 | 66 | 5.7 |
| Rarely | 7 | 4.3 | 24 | 5.6 | 21 | 5.0 | 11 | 7.1 | 63 | 5.4 |
| Sometimes | 36 | 22.4 | 102 | 23.9 | 89 | 21.3 | 33 | 21.3 | 260 | 22.4 |
| Often | 22 | 13.7 | 78 | 18.3 | 79 | 18.9 | 45 | 29.0 | 224 | 19.3 |
| Always | 92 | 57.1 | 200 | 46.9 | 198 | 47.5 | 56 | 36.1 | 546 | 47.1 |

**Supplementary Table 3**. Information needs concerning the effects of specific diets, foods, nutrients and dietary supplements in managing cancer treatment side effects.

(N = number of respondents, % = percentage of respondents).

|  | **Overall**  **(N=1159)** | |
| --- | --- | --- |
|  | **N** | **%** |
| **Changes in taste** |  |  |
| Not at all | 269 | 23.2 |
| Slightly | 198 | 17.1 |
| Moderately | 307 | 26.5 |
| Quite a bit | 229 | 19.8 |
| Extremely | 156 | 13.5 |
| **Changes in lipid profile** |  |  |
| Not at all | 114 | 9.8 |
| Slightly | 115 | 9.9 |
| Moderately | 348 | 30.0 |
| Quite a bit | 327 | 28.2 |
| Extremely | 255 | 22.0 |
| **Weight gain** |  |  |
| Not at all | 77 | 6.6 |
| Slightly | 95 | 8.2 |
| Moderately | 303 | 26.1 |
| Quite a bit | 352 | 30.4 |
| Extremely | 332 | 28.6 |
| **Diarrhea** |  |  |
| Not at all | 325 | 28.0 |
| Slightly | 205 | 17.7 |
| Moderately | 264 | 22.8 |
| Quite a bit | 209 | 18.0 |
| Extremely | 156 | 13.5 |
| **Dehydration** |  |  |
| Not at all | 231 | 19.9 |
| Slightly | 194 | 16.7 |
| Moderately | 292 | 25.2 |
| Quite a bit | 264 | 22.8 |
| Extremely | 178 | 15.4 |
| **Fatigue** |  |  |
| Not at all | 118 | 10.2 |
| Slightly | 113 | 9.7 |
| Moderately | 297 | 25.6 |
| Quite a bit | 321 | 27.7 |
| Extremely | 310 | 26.7 |
| **Gastritis** |  |  |
| Not at all | 266 | 23.0 |
| Slightly | 201 | 17.3 |
| Moderately | 261 | 22.5 |
| Quite a bit | 249 | 21.5 |
| Extremely | 182 | 15.7 |
| **Abdominal swelling** |  |  |
| Not at all | 126 | 10.9 |
| Slightly | 130 | 11.2 |
| Moderately | 313 | 27.0 |
| Quite a bit | 321 | 27.7 |
| Extremely | 269 | 23.2 |
| **Subcutaneous swelling/edema** |  |  |
| Not at all | 275 | 23.7 |
| Slightly | 176 | 15.2 |
| Moderately | 251 | 21.7 |
| Quite a bit | 252 | 21.7 |
| Extremely | 205 | 17.7 |
| **Nausea and vomiting** |  |  |
| Not at all | 337 | 29.1 |
| Slightly | 192 | 16.6 |
| Moderately | 231 | 19.9 |
| Quite a bit | 223 | 19.2 |
| Extremely | 176 | 15.2 |
| **Osteoporosis** |  |  |
| Not at all | 76 | 6.6 |
| Slightly | 84 | 7.2 |
| Moderately | 284 | 24.5 |
| Quite a bit | 329 | 28.4 |
| Extremely | 386 | 33.3 |
| **Loss of appetite** |  |  |
| Not at all | 420 | 36.2 |
| Slightly | 201 | 17.3 |
| Moderately | 219 | 18.9 |
| Quite a bit | 188 | 16.2 |
| Extremely | 131 | 11.3 |
| **Cognitive dysfunction** |  |  |
| Not at all | 100 | 8.6 |
| Slightly | 114 | 9.8 |
| Moderately | 269 | 23.2 |
| Quite a bit | 300 | 25.9 |
| Extremely | 376 | 32.4 |
| **Dry mouth** |  |  |
| Not at all | 272 | 23.5 |
| Slightly | 195 | 16.8 |
| Moderately | 259 | 22.3 |
| Quite a bit | 228 | 19.7 |
| Extremely | 205 | 17.7 |
| **Constipation** |  |  |
| Not at all | 258 | 22.3 |
| Slightly | 190 | 16.4 |
| Moderately | 259 | 22.3 |
| Quite a bit | 235 | 20.3 |
| Extremely | 217 | 18.7 |
| **Stomatitis** |  |  |
| Not at all | 382 | 33.0 |
| Slightly | 185 | 16.0 |
| Moderately | 234 | 20.2 |
| Quite a bit | 199 | 17.2 |
| Extremely | 159 | 13.7 |

**Supplementary Table 4**. Information needs concerning the use of specific diets, in oncology.

(N = number of respondents, % = percentage of respondents).

|  | **Overall**  **(N=1159)** | |
| --- | --- | --- |
|  | **N** | **%** |
| **Mediterranean diet** |  |  |
| Not at all | 33 | 2.8 |
| Slightly | 86 | 7.4 |
| Moderately | 365 | 31.5 |
| Quite a bit | 387 | 33.4 |
| Extremely | 288 | 24.8 |
| **Vegetarian diet** |  |  |
| Not at all | 220 | 19.0 |
| Slightly | 191 | 16.5 |
| Moderately | 294 | 25.4 |
| Quite a bit | 279 | 24.1 |
| Extremely | 175 | 15.1 |
| **Vegan diet** |  |  |
| Not at all | 486 | 41.9 |
| Slightly | 225 | 19.4 |
| Moderately | 191 | 16.5 |
| Quite a bit | 143 | 12.3 |
| Extremely | 114 | 9.8 |
| **Macrobiotic diet** |  |  |
| Not at all | 527 | 45.5 |
| Slightly | 275 | 23.7 |
| Moderately | 182 | 15.7 |
| Quite a bit | 113 | 9.7 |
| Extremely | 62 | 5.3 |
| **High-protein diet** |  |  |
| Not at all | 531 | 45.8 |
| Slightly | 268 | 23.1 |
| Moderately | 200 | 17.3 |
| Quite a bit | 103 | 8.9 |
| Extremely | 57 | 4.9 |
| **High-calorie diet** |  |  |
| Not at all | 593 | 51.2 |
| Slightly | 269 | 23.2 |
| Moderately | 160 | 13.8 |
| Quite a bit | 85 | 7.3 |
| Extremely | 52 | 4.5 |
| **Ketogenic diet** |  |  |
| Not at all | 565 | 48.7 |
| Slightly | 236 | 20.4 |
| Moderately | 182 | 15.7 |
| Quite a bit | 115 | 9.9 |
| Extremely | 61 | 5.3 |
| **Blood-type diet** |  |  |
| Not at all | 628 | 54.2 |
| Slightly | 199 | 17.2 |
| Moderately | 160 | 13.8 |
| Quite a bit | 111 | 9.6 |
| Extremely | 61 | 5.3 |
| **Acid-alkaline diet** |  |  |
| Not at all | 622 | 53.7 |
| Slightly | 215 | 18.6 |
| Moderately | 161 | 13.9 |
| Quite a bit | 96 | 8.3 |
| Extremely | 65 | 5.6 |
| **Low-glycemic index diet** |  |  |
| Not at all | 490 | 42.3 |
| Slightly | 221 | 19.1 |
| Moderately | 211 | 18.2 |
| Quite a bit | 146 | 12.6 |
| Extremely | 91 | 7.9 |
| **Paleo diet** |  |  |
| Not at all | 721 | 62.2 |
| Slightly | 222 | 19.2 |
| Moderately | 107 | 9.2 |
| Quite a bit | 69 | 6.0 |
| Extremely | 40 | 3.5 |
| **Raw food diet** |  |  |
| Not at all | 763 | 65.8 |
| Slightly | 189 | 16.3 |
| Moderately | 109 | 9.4 |
| Quite a bit | 60 | 5.2 |
| Extremely | 38 | 3.3 |
| **Detox diet/Cleansing diet** |  |  |
| Not at all | 556 | 48.0 |
| Slightly | 194 | 16.7 |
| Moderately | 198 | 17.1 |
| Quite a bit | 126 | 10.9 |
| Extremely | 85 | 7.3 |
| **Fasting** |  |  |
| Not at all | 429 | 37.0 |
| Slightly | 212 | 18.3 |
| Moderately | 221 | 19.1 |
| Quite a bit | 148 | 12.8 |
| Extremely | 149 | 12.9 |
| **Dietary restrictions** |  |  |
| Not at all | 405 | 34.9 |
| Slightly | 200 | 17.3 |
| Moderately | 242 | 20.9 |
| Quite a bit | 179 | 15.4 |
| Extremely | 133 | 11.5 |

**Supplementary Table 5**. Information needs concerning the use of some foods and nutrients, in oncology.

(N = number of respondents, % = percentage of respondents).

|  | **Overall**  **(N=1159)** | |
| --- | --- | --- |
|  | **N** | **%** |
| **Soy and soy products** |  |  |
| Not at all | 142 | 12.3 |
| Slightly | 164 | 14.2 |
| Moderately | 355 | 30.6 |
| Quite a bit | 294 | 25.4 |
| Extremely | 204 | 17.6 |
| **Milk and dairy products** |  |  |
| Not at all | 52 | 4.5 |
| Slightly | 82 | 7.1 |
| Moderately | 383 | 33.0 |
| Quite a bit | 376 | 32.4 |
| Extremely | 266 | 23.0 |
| **Sugars** |  |  |
| Not at all | 57 | 4.9 |
| Slightly | 70 | 6.0 |
| Moderately | 355 | 30.6 |
| Quite a bit | 359 | 31.0 |
| Extremely | 318 | 27.4 |
| **Refined flours** |  |  |
| Not at all | 68 | 5.9 |
| Slightly | 84 | 7.2 |
| Moderately | 368 | 31.8 |
| Quite a bit | 362 | 31.2 |
| Extremely | 277 | 23.9 |
| **Red and processed meat** |  |  |
| Not at all | 113 | 9.7 |
| Slightly | 99 | 8.5 |
| Moderately | 344 | 29.7 |
| Quite a bit | 344 | 29.7 |
| Extremely | 259 | 22.3 |
| **White meat** |  |  |
| Not at all | 91 | 7.9 |
| Slightly | 96 | 8.3 |
| Moderately | 397 | 34.3 |
| Quite a bit | 351 | 30.3 |
| Extremely | 224 | 19.3 |
| **Alcoholic beverages** |  |  |
| Not at all | 336 | 29.0 |
| Slightly | 140 | 12.1 |
| Moderately | 273 | 23.6 |
| Quite a bit | 233 | 20.1 |
| Extremely | 177 | 15.3 |
| **Pulses** |  |  |
| Not at all | 73 | 6.3 |
| Slightly | 82 | 7.1 |
| Moderately | 394 | 34.0 |
| Quite a bit | 366 | 31.6 |
| Extremely | 244 | 21.1 |
| **Citruses** |  |  |
| Not at all | 76 | 6.6 |
| Slightly | 103 | 8.9 |
| Moderately | 382 | 33.0 |
| Quite a bit | 374 | 32.3 |
| Extremely | 224 | 19.3 |
| **Solanaceae (aubergines, tomatoes, potatoes)** |  |  |
| Not at all | 61 | 5.3 |
| Slightly | 90 | 7.8 |
| Moderately | 365 | 31.5 |
| Quite a bit | 388 | 33.5 |
| Extremely | 255 | 22.0 |
| **Carbohydrates** |  |  |
| Not at all | 35 | 3.0 |
| Slightly | 95 | 8.2 |
| Moderately | 362 | 31.2 |
| Quite a bit | 384 | 33.1 |
| Extremely | 283 | 24.4 |
| **Fats** |  |  |
| Not at all | 53 | 4.6 |
| Slightly | 108 | 9.3 |
| Moderately | 367 | 31.7 |
| Quite a bit | 366 | 31.6 |
| Extremely | 265 | 22.9 |
| **Proteins** |  |  |
| Not at all | 37 | 3.2 |
| Slightly | 92 | 7.9 |
| Moderately | 365 | 31.5 |
| Quite a bit | 391 | 33.7 |
| Extremely | 274 | 23.6 |

**Supplementary Table 6**. Information needs concerning the use of dietary supplements, in oncology.

(N = number of respondents, % = percentage of respondents).

|  | **Overall**  **(N=1159)** | |
| --- | --- | --- |
|  | **N** | **%** |
| **Vitamins and mineral salts** |  |  |
| Not at all | 49 | 4.2 |
| Slightly | 85 | 7.3 |
| Moderately | 410 | 35.4 |
| Quite a bit | 356 | 30.7 |
| Extremely | 259 | 22.3 |
| **Alternative medicine** |  |  |
| Not at all | 289 | 24.9 |
| Slightly | 206 | 17.8 |
| Moderately | 277 | 23.9 |
| Quite a bit | 238 | 20.5 |
| Extremely | 149 | 12.9 |
| **Herbal dietary supplements** |  |  |
| Not at all | 159 | 13.7 |
| Slightly | 182 | 15.7 |
| Moderately | 337 | 29.1 |
| Quite a bit | 291 | 25.1 |
| Extremely | 190 | 16.4 |
| **Homeopathic products** |  |  |
| Not at all | 325 | 28.0 |
| Slightly | 195 | 16.8 |
| Moderately | 266 | 23.0 |
| Quite a bit | 227 | 19.6 |
| Extremely | 146 | 12.6 |
| **Lactic cultures/probiotics** |  |  |
| Not at all | 101 | 8.7 |
| Slightly | 145 | 12.5 |
| Moderately | 377 | 32.5 |
| Quite a bit | 310 | 26.7 |
| Extremely | 226 | 19.5 |
| **Omega-3, EPA/DHA** |  |  |
| Not at all | 131 | 11.3 |
| Slightly | 137 | 11.8 |
| Moderately | 355 | 30.6 |
| Quite a bit | 314 | 27.1 |
| Extremely | 222 | 19.2 |
| **Turmeric and ginger** |  |  |
| Not at all | 182 | 15.7 |
| Slightly | 160 | 13.8 |
| Moderately | 327 | 28.2 |
| Quite a bit | 287 | 24.8 |
| Extremely | 203 | 17.5 |

**Supplementary Figure 1**. Instagram live broadcasts engagement rates (preset timeframe “Last 90 days”). (A) Instagram live broadcast 1: “Nutrition and breast cancer, what would you like to know? Diets”; (B) Instagram live broadcast 2: “Nutrition and breast cancer, what would you like to know? Foods and nutrients”; (C) Instagram live broadcast 3: “Nutrition and breast cancer, what would you like to know? Dietary supplements”; (D) Instagram live broadcast 4: “Nutrition and breast cancer, what would you like to know? Intolerance of uncertainty”.

Ottobre: October; Durata: length.


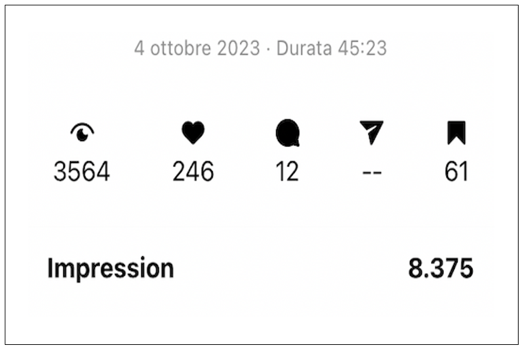

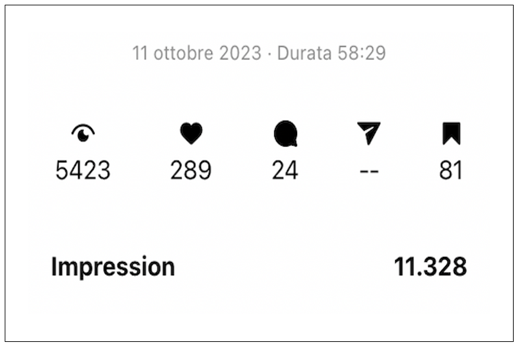


A B


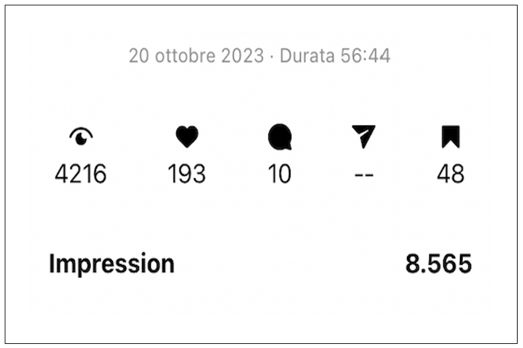

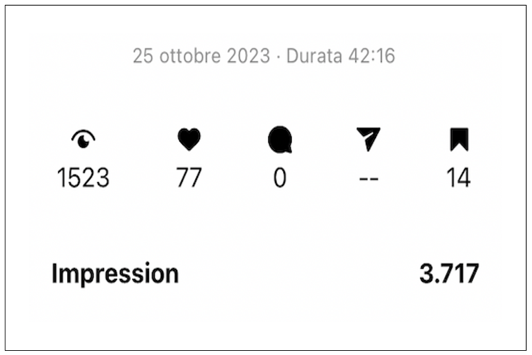


C D
